# Rhamnolipids from *Pseudomonas aeruginosa* disperse the biofilms of sulfate-reducing bacteria

**DOI:** 10.1038/s41522-018-0066-1

**Published:** 2018-10-03

**Authors:** Thammajun L. Wood, Ting Gong, Lei Zhu, James Miller, Daniel S. Miller, Bei Yin, Thomas K. Wood

**Affiliations:** 1 Department of Chemical Engineering, Pennsylvania State University, University Park, PA 16802 USA; 2 Huck Institutes of the Life Sciences, Pennsylvania State University, University Park, PA 16802 USA; 3 Dow Chemical Company, Collegeville, PA 19426 USA; 4 Department of Biochemistry and Molecular Biology, Pennsylvania State University, University Park, PA 16802 USA

## Abstract

Biofilm formation is an important problem for many industries. *Desulfovibrio vulgaris* is the representative sulfate-reducing bacterium (SRB) which causes metal corrosion in oil wells and drilling equipment, and the corrosion is related to its biofilm formation. Biofilms are extremely difficult to remove since the cells are cemented in a polymer matrix. In an effort to eliminate SRB biofilms, we examined the ability of supernatants from *Pseudomonas aeruginosa* PA14 to disperse SRB biofilms. We found that the *P*. *aeruginosa* supernatants dispersed more than 98% of the biofilm. To determine the biochemical basis of this SRB biofilm dispersal, we examined a series of *P*. *aeruginosa* mutants and found that mutants *rhlA*, *rhlB*, *rhlI*, and *rhlR*, defective in rhamnolipids production, had significantly reduced levels of SRB biofilm dispersal. Corroborating these results, purified rhamnolipids dispersed SRB biofilms, and rhamnolipids were detected in the *P*. *aeruginosa* supernatants. Hence, *P*. *aeruginosa* supernatants disperse SRB biofilms via rhamnolipids. To determine the genetic basis of how the *P*. *aeruginosa* supernatants disperse SRB biofilms, a whole transcriptomic analysis was conducted (RNA-seq); based on this analysis, we identified four proteins (DVUA0018, DVUA0034, DVUA0066, and DVUA0084) of the *D*. *vulgaris* megaplasmid that influence biofilm formation, with production of DVUA0066 (a putative phospholipase) reducing biofilm formation 5.6-fold. In addition, the supernatants of *P*. *aeruginosa* dispersed the SRB biofilms more readily than protease in M9 glucose minimum medium and were also effective against biofilms of *Escherichia coli* and *Staphylococcus aureus*.

## Industrial implications: Breaking down corrosive biofilms

A subclass of glycolipids produced by the bacterium *Pseudomonas aeruginosa* breaks down a corrosive bacterial biofilm that afflicts industries such as petroleum extraction. The *P*. *aeruginosa* molecules, known as rhamnolipids, have been previously used to break down other bacterial biofilms. Now, a team from the United States, led by Pennsylvania State University’s Thomas Wood, showed that extracts from *P*. *aeruginosa* dispersed 98% of *Desulfovibrio vulgaris* biofilm, which is problematic for many industries, and also dispersed *Escherichia coli* and *Staphylococcus aureus* biofilms. Genetic investigations showed rhamnolipids were responsible for the majority of the biofilm inhibition. Further analyses also revealed four *D*. *vulgaris* proteins that significantly affect biofilm formation. This research highlights a mechanism that may be used to reduce the impact of industry-affecting biofilms and deepens the understandings of the mechanisms of biofilm formation.

## Introduction

Sulfate-reducing bacteria (SRB) are an important type of microorganism causing iron corrosion on metal surfaces under both anaerobic and aerobic conditions.^1–3^ *Desulfovibrio vulgaris* Hildenborough is a sequenced^4^ Gram-negative SRB that has been used as an SRB model organism to study biocorrosion and bioremediation of toxic metal ions^4^ as well as biofilm formation^5,6^ and bioimmobilization at superfund sites.^7^ It is also called the “petroleum pest” because it is commonly found in oil fields and causes “souring” of petroleum and damage to topside equipment and pipelines.^8^

Biofilms are groups of bacteria that are held together in a self-produced extracellular matrix^9^ and are difficult to remove with antimicrobial agents due to their antibiotic or biocide resistance relative to planktonic cells.^10^ *Desulfovibrio* sp. populations in biofilms have a significant role for microbial induced corrosion because of their sulfide production and electron transfer mechanism,^5^ and biofilms of *D*. *vulgaris* have been extensively shown to cause corrosion in many types of steels and other alloys.^11^ The biofilms of *D*. *vulgaris* consists primarily of protein,^5^ mannose,^6^ fucose,^6^ and *N*-acetylgalactosamine.^6^ The biofilms of *Escherichia coli* consist primarily of proteinaceous curli fibres, flagella, and the polysaccharide cellulose,^12^ and the biofilms of *Staphylococcus aureus* are largely composed of cytoplasmic proteins^13^ and extracellular genomic DNA.^14^

Many Gram-negative bacteria use quorum sensing (QS) molecules or autoinducers to communicate with each other^15^ and to form biofilms.^15^ The QS mechanism can control particular processes related to cell density,^16^ and QS inhibition targeting autoinducers has been used as a method to control biofilm formation.^16^ The opportunistic pathogen *Pseudomonas aeruginosa* has four QS systems (Las, Rhl, Pqs, and Iqs).^17^ Each QS system has its own signal and regulatory protein. For the Las system, LasI synthesizes *N*-(3-oxododecanoyl)-homoserine lactone (3oxoC12HSL), and LasR is the protein receptor.^17^ For the Rhl system, RhlI synthesizes *N*-butyrylhomoserine lactone (C4-HSL), and RhlR is the protein receptor. The third QS system is quinolone-based intercellular signaling; the PQS signal is 2-heptyl-3-hydroxy-4-quinolone, and it is synthesized by the products of the PQS synthesis cluster consisting of *pqsABCD*, *phnAB*, and *pqsH*. PqsR is the protein receptor.^17^ The fourth system is called Iqs, and the QS molecule is 2-(2-hydroxyphenyl)-thiazole-4-carbaldehyde.^17^

One aspect of biofilm formation controlled by the Rhl QS system is regulation of the synthesis of rhamnolipids, which are glycolipid biosurfactants composed of rhamnose and 3-(hydroxyalkanoyloxy) alkanoic acid (HAA).^18^ *P*. *aeruginosa* rhamnolipids affect biofilm architecture by participating in the maintenance of biofilm channels^19^ and by reducing adhesion between cells^20^; hence, they have been used to disperse biofilms of *Bordetella bronchiseptica*,^21^ *Bacillus pumilus*,^22^ *Staphylococcus aureus*,^23^ *Listeria monocytogenes*^20^ and *Salmonella enteritidis*.^20^ In addition, rhamnolipids from *Lysinibacillus* sp. BV152.1 also inhibit biofilm formation of *P*. *aeruginosa* PAO1, *S*. *aureus*, and *Serratia marcescens*.^24^

There are many reasons for forming bacterial biofilms such as a defense against stress (e.g., nutrient deprivation, antibiotics, or pH changes)^25^ and a mechanism for staying stationary in a nutrient-rich area.^25^ However, bacteria also must have a means to leave the biofilm (dispersal) due to environmental changes (e.g., fluctuations in oxygen levels, alterations in nutrients, or increasing of toxic products)^26^; this process involves breaking the matrix to uncement the cells.^27^

Here, we show that supernatants from *P*. *aeruginosa* PA14 (henceforth PA14) disperse the biofilms of SRB, *E*. *coli*, and *S*. *aureus*. Using QS-related mutants, we determined that the biochemical basis for this dispersal is the presence of rhamnolipids in the supernatants. We also investigated the genetic basis of dispersal of *D*. *vulgaris* biofilms via PA14 supernatants by RNA sequencing (RNA-seq) and found that DVUA0018, DVUA0034, DVUA0066, and DVUA0084 encoded by the *D*. *vulgaris* megaplasmid are related to biofilm formation.

## Results

### PA14 wild-type supernatant disperses SRB biofilm

**Fig. 1:**
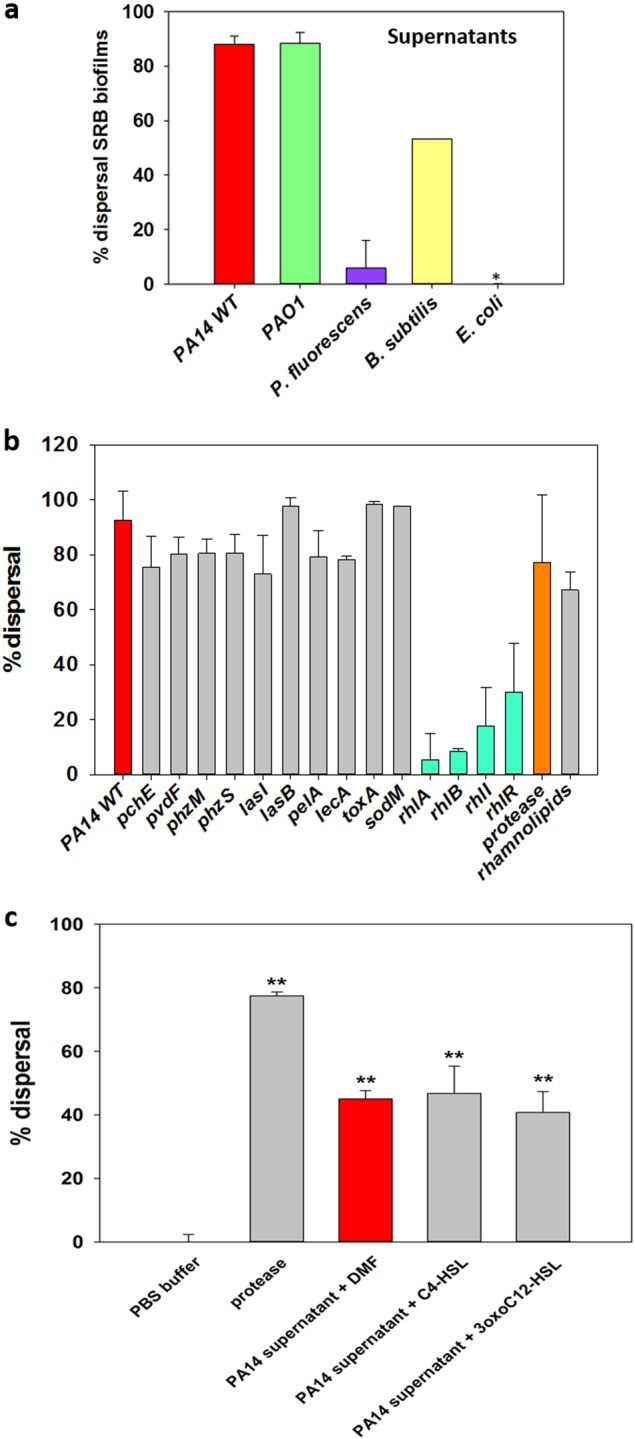
*D*. *vulgaris* biofilm dispersal by supernatants of *P*. *aeruginosa* PA14, its quorum sensing mutants, *P*. *aeruginosa* PAO1, *B*. *subtilis*, *P*. *fluorescens*, *E*. *coli* TG1, and homoserine lactones. (**a**) *D*. *vulgaris* biofilms were grown for 2 days in modified Baar’s media at 30 °C, and all of the supernatants were concentrated to 4× and contacted with *D*. *vulgaris* biofilms for 2 h. * indicates no dispersal. At least two independent cultures were used with three replicates for each culture, and the error bars indicate one standard deviation. (**b**) *D*. *vulgaris* biofilms were grown for 2 days in modified Baar’s medium at 30 °C, and supernatants were concentrated to 4× and contacted with *D*. *vulgaris* biofilms for 2 h. Rhamnolipid standards were added at 10 mM. Protease 1 (Savinase) at 0.024 U was used as a positive control. At least two independent cultures were used with three replicates for each culture, and the error bars indicate one standard deviation. (**c**) *D*. *vulgaris* was grown for 24 h to form biofilms in modified Baar’s medium. PA14 wild-type supernatants were used at 1× (so that the effect of the homoserine lactones could be more clearly discerned) with 50 µM of C4-HSL, 3oxoC12HSL, or DMF (negative control) added for 90 min. Savinase (protease) at 0.024 U was also used as a positive control with PBS buffer used as negative control for protease. Two independent cultures and three replicate wells for each condition were used. The error bar indicates one standard deviation. The symbols ** (*P* < 0.01) indicate significant differences versus the PA14 supernatants + DMF control via one-way ANOVA

In an effort to investigate whether there are QS compounds utilized by the representative SRB *D*. *vulgaris*, we tested whether its supernatants would disperse its own biofilm formed in rich medium. *D*. *vulgaris* supernatant did not disperse its own biofilm within 2 h (data not shown). Since there was no negative effect of SRB supernatants on its own biofilm, we investigated the effect of the supernatant of other species (e.g., *P*. *aeruginosa*, *P*. *fluorescens*, *Bacillus subtilis*, *E*. *coli*). Among these species, the supernatants (concentrated 4×) of *P*. *aeruginosa* PA14 and *P*. *aeruginosa* PAO1 dispersed SRB biofilm the most (Fig. 1a). The supernatants were obtained from planktonic stationary-phase cultures, and the SRB biofilm grown was in 96-well plates for 24–48 h in modified Baar’s medium. Critically, the PA14 wild-type supernatant dispersed *D*. *vulgaris* biofilm more than 92% after 1–2 h of incubation. We utilized short periods of contact of the supernatants with the SRB biofilm to avoid artifacts related with growth of the bacterium.

### Rhamnolipids in the supernatants disperse SRB biofilms

To determine mechanisms behind these strong dispersal results, we hypothesized that the compounds in the *P*. *aeruginosa* supernatant may be related to QS since PA14 is the best known strain for QS, and QS controls extracellular compounds like protease^28^ and rhamnolipids.^28^ Hence, we tested the supernatant of mutants related to QS as well as those related to virulence factors (e.g., *lasI*,^29^ *lasB*,^30^ *pelA*,^31^ *phzM*,^32^ *phzS*,^32^ *pvdF*,^33^ *pchE*,^34^ *lecA*,^35^ *toxA*,^36^ *sodM*,^37^ *rhlI*,^38^ and *rhlR*^38^). Similar to the supernatant of the PA14 wild-type, the supernatants of the mutants *lasI* (autoinducer synthase), *lasB* (elastase), *pelA* (oligogalacturonide lyase), *phzM* (pyocyanin biosynthesis), *phzS* (pyocyanin biosynthesis), *pvdF* (pyoverdin biosynthesis), *pchE* (pyochelin biosynthesis), *lecA* (LecA lectin), *toxA* (exotoxinA), and *sodM* (superoxide dismutase) dispersed the *D*. *vulgaris* biofilm like the supernatant of PA14 wild-type (Fig. 1b), indicating the Las QS system, Pel polysaccharide, pyocyanin, pyoverdine, pyocheline, LecA lectin, exotoxin A, superoxide dismutase had no role in the biofilm dispersal. To corroborate these QS results, we added both C4-HSL and 3oxoC12HSL to the wild-type supernatants without concentration; i.e., 1×, so the effect of the homoserine lactones could be more clearly discerned, but there was no additional dispersal of the SRB biofilm (Fig. 1c).

In contrast to the other mutants, mutations affecting the regulation and biosynthesis of rhamnolipids, specifically in the gene encoding the autoinducer synthase, *rhlI*, and the transcriptional regulator, *rhlR*, had a pronounced decrease in dispersal (Fig. 1b). Since the *rhlI* and *rhlR* mutants were found to affect biofilm dispersal, additional mutations in the rhamnolipid pathway were investigated: *rhlA*, which encodes rhamnosyltransferse 1 subunit A and *rhlB*, which encodes the catalytic subunit of the rhamnosyltransferase.^38,39^ The supernatants of both the *rhlA* and *rhlB* mutant did not disperse SRB biofilms (Fig. 1b). Therefore, the dispersal compounds are related to rhamnolipids.

**Fig. 2:**
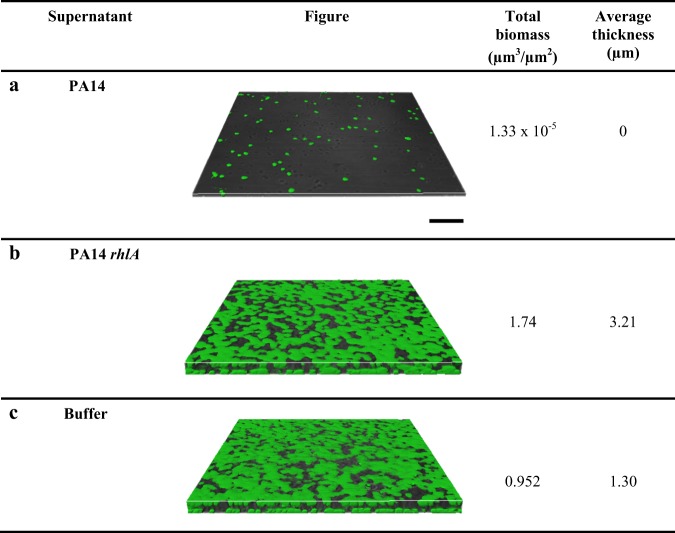
Representative images of *D*. *vulgaris* biofilm dispersal via *P*. *aeruginosa* supernatants as visualized by confocal microscopy. *D*. *vulgaris* biofilms were formed in an 8 chamber cell culture slide for 48 h in modified Baar’s medium. Supernatants were concentrated to 4× and contacted with *D*. *vulgaris* biofilms for 20 min: supernatants of (**a**) *P*. *aeruginosa* PA14, (**b**) *P*. *aeruginosa* PA14 *rhlA*, and (**c**) PBS buffer (used as a negative control to indicate what undispersed biofilms after the removal planktonic cells and loose biofilm). Scale bar is 50 µm

To corroborate the 96-well biofilm dispersal results with the PA14 wild-type and *rhlA* mutant, confocal microscopy was used to visualize the remaining biofilm after treatment with supernatants from these two strains. As shown in Fig. 2, the supernatant from the wild-type strain nearly completely dispersed the SRB biofilm whereas the supernatant from the *rhlA* mutant had no effect, just like the buffer negative control.

**Fig. 3:**
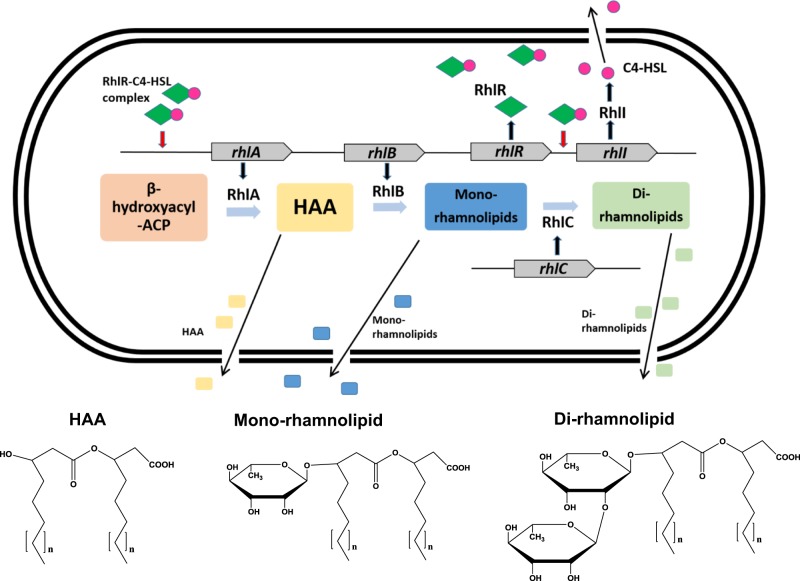
Simplified rhamnolipid biosynthesis pathway in *P*. *aeruginosa*.^56,57^ RhlR binds with *N*-butanoyl-L-homoserine lactone (C4-HSL) produced by RhlI to form a RhlR–C4-HSL complex. The RhlR–C4-HSL complex interacts with the *rhlA* promoter to initiate transcription of the *rhlAB* genes to produce rhamnolipids. HAA is 3-(hydroxyalkanoyloxy) alkanoic acid. *P*. *aeruginosa* commonly produces rhamnolipids containing fatty acids with chain lengths between C_8_ and C_14_ (*n* = 1–7).^58^

From the rhamnolipid production pathway (Fig. 3), RhlA synthesizes 3-(3-hydroxyakanoyloxy) alkanoic acids (HAA),^40^and RhlB uses HAA to make mono-rhamnolipids.^41^ Therefore, the compounds required for SRB biofilm dispersal are HAA or mono/di-rhamnolipids, respectively. However, the results from mass spectrometry showed that there was no HAA detected in the wild-type supernatant sample. Therefore we conclude that rhamnolipids are the compounds (and not HAA) that are important for biofilm dispersal.

### Rhamnolipids in the PA14 supernatants disperse SRB biofilms

The genetic results clearly indicate the importance of rhamnolipids for SRB biofilm dispersal via PA14 supernatants. To corroborate these results, the 4× concentrated supernatants of PA14, *rhlA*, *rhlB*, *rhlI*, and *rhlR* mutants were tested for the presence of rhamnolipids by mass spectrometry (Supplementary Fig. 1). As expected, rhamnolipids were detected only in the PA14 supernatant samples and not from any of the mutant samples. There were four predominant peaks of rhamnolipids shown (Supplementary Fig. 1) which are Rha-C_10_–C_10_, Rha-C_10_–C_12_/Rha-C_12_–C_10_, Rha-Rha-C_10_–C_10_, and Rha-Rha-C_10_–C_12_/ Rha-Rha-C_12_–C_10_. By comparing with the 10 mM rhamnolipids standard from *P*. *aeruginosa*, we determined the concentration of rhamnolipids in the PA14 supernatant using the total peak areas is over 100 fold less than the standard (0.1 mM, Supplementary Table 1).

To show conclusively that rhamnolipids in the supernatants are the biochemical means by which the *D*. *vulgaris* biofilms are dispersed, commercial, purified *P*. *aeruginosa* rhamnolipids (10 mM) were tested. We found that the commercial rhamnolipids disperse *D*. *vulgaris* biofilm with approximately 67% dispersal after 2 h (Fig. 1b). The commercial rhamnolipids standard did not disperse the biofilm as well as the PA14 supernatant (90%, Fig. 1b) though there were 100× more rhamnolipids in the 10 mM commercial standard sample than the amount of rhamnolipids in the supernatant (Supplementary Table 1). This implies that the ratio of the rhamnolipids or other compounds in the supernatant are important for SRB biofilm dispersal; the commercial rhamnolipids may also be altered upon purification. We tested whether other components in the supernatant were required by adding the supernatant of the *rhlB* mutant to the rhamnolipids commercial standard, but there was no increase in biofilm dispersal compared to the rhamnolipids standard alone. This suggests the dispersal is due to the rhamnolipids alone and not some other product in the supernatants.

We also tested whether PA14 supernatants decrease the viability of SRB planktonic cells and found their viability is reduced by 60 ± 8% with wild-type supernatants (5×) and 37 ± 2% by *rhlA* supernatants (5×, that lack rhamnolipids). The purified commercial rhamnolipids (10 mM) reduced planktonic SRB viability by 17.0 ± 0.6%. These results corroborate the previous report that rhamnolipids from *P*. *aeruginosa* PA14 have some toxicity^42^ and indicate there are other compounds with toxicity in the supernatants. Critically, we tested the effect of PA14 supernatants (concentrated 4×, to match the earlier dispersal conditions) on SRB biofilm cells (which mirrors the conditions used in this manuscript to disperse biofilm cells) and found significantly less toxicity for both wild-type (23 ± 3%) and *rhlA* supernatants (22.4 ± 0.5%); the purified commercial rhamnolipids reduced viability by 9 ± 2%.

### PA14 supernatants disperse *D*. *desulfuricans*, *E*. *coli* MG1655, and *Staphylococcus aureus* biofilms

To test the ability of the PA14 supernatants to disperse other bacteria, biofilms of *D*. *desulfuricans*, *E*. *coli* MG1655, *S*. *aureus*, and PA14 were grown in 96-well plates, then treated with supernatants from PA14. The PA14 supernatant dispersed the biofilms of *D*. *desulfuricans*, *E*. *coli* MG1655, and *S*. *aureus* well (by over 90%). Although, there are some species-specific differences, these results show that the PA14 supernatants have a great deal of utility for dispersing a wide range of biofilms.

**Fig. 4:**
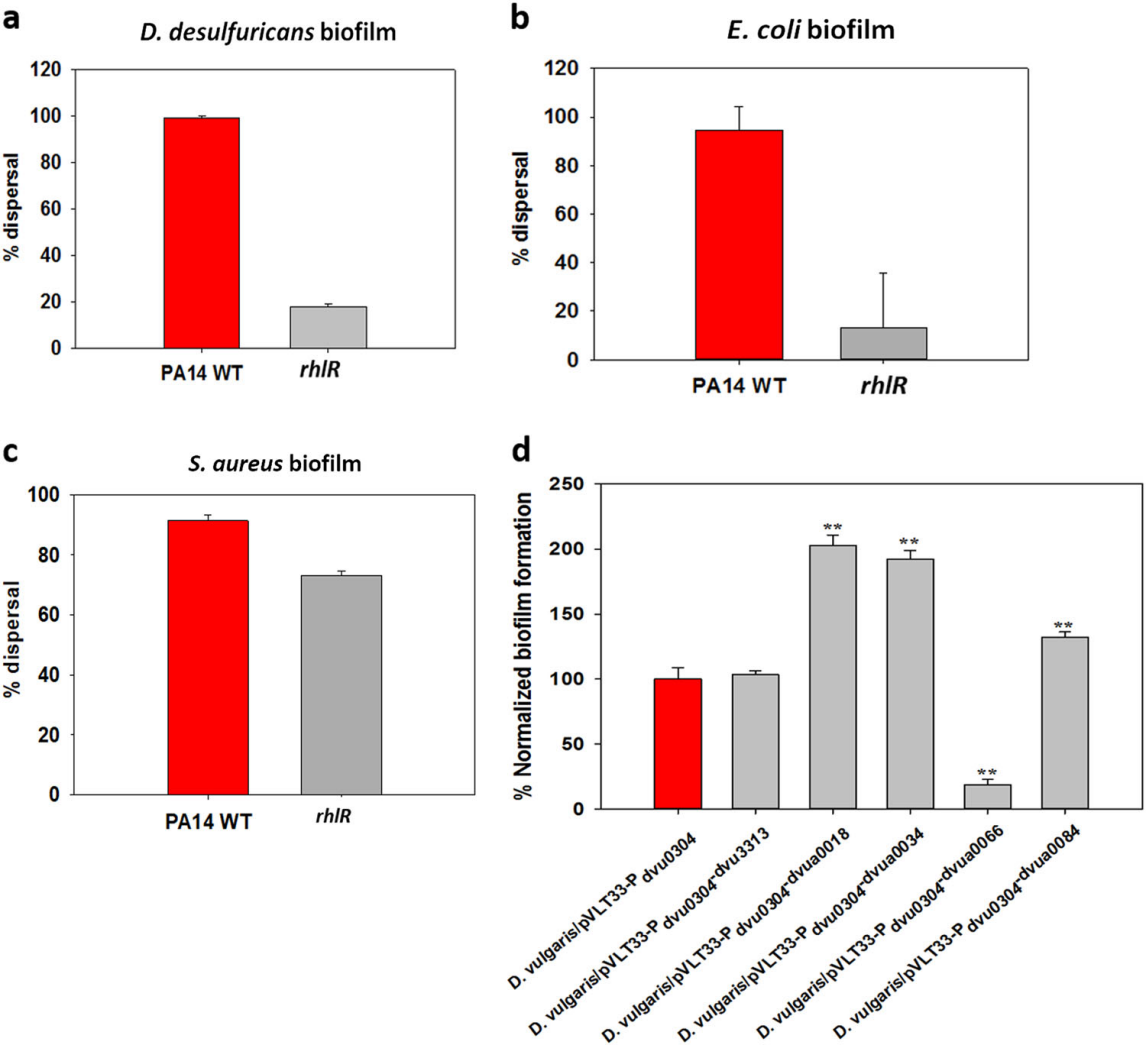
Supernatants of *P*. *aeruginosa* PA14 disperse diverse biofilms, and *D*. *vulgaris* biofilm formation during production of putative dispersal proteins identified through RNA-seq. The biofilms of *D*. *desulfuricans* (**a**) and *E*. *coli* MG1655 (**b**) were grown for 24 h in modified Baar’s medium and LB, respectively, and the biofilms of *S*. *aureus* (**c**) were grown for 24 h in TSB.^59^ *P*. *aeruginosa* wild-type and *rhlR* mutant supernatants were concentrated to 4× and contacted with the biofilms for 2 h except for the *S*. *aureus* biofilm where 1x supernatant was used instead of 4× and the contact time was 10 min (since *S*. *aureus* biofilms were dispersed quickly). At least two independent cultures were used with three replicates for each culture, and the error bars indicate one standard deviation. (**d**) Biofilms of *D*. *vulgaris* were formed for 48 h in modified Baar’s medium at 30 °C (anaerobically) with expression of *dvu3313*, *dvua0018*, *dvua0034*, *dvua0066*, and *dvua0084* identified from the dispersal RNA-seq experiment. Vector pVLT33 was used with the biofilm-specific promoter P_*dvu0304*_ fused to each gene. Normalized biofilm formation percentage (based on OD_540nm_/OD_620nm_) is shown relative to the empty plasmid control. Two independent cultures were used with three replicates for each culture, and the error bars indicate one standard deviation. ** *P* < 0.01 indicates significant differences versus the empty plasmid control group via one-way ANOVA

The supernatant from the *rhlR* mutant did not disperse the *D*. *desulfuricans* and *E*. *coli* biofilms well (Fig. 4), providing further evidence of the importance of rhamnolipids for these species. However, there is a rhamnolipid-independent component responsible for dispersing *S*. *aureus* biofilms. In addition, the supernatant of PA14 did not disperse its own biofilm (data not shown).

### PA14 supernatant disperses SRB biofilm grown in M9G

Since biofilm matrices can change when the growth medium is altered,^43^ we tested the ability of the PA14 supernatant to disperse SRB biofilms grown in minimal glucose medium (M9G). The PA14 supernatant (4x concentrated) dispersed 90–100% of the SRB biofilm when formed in modified Baar’s medium and dispersed about 80% of the biofilm formed in M9G. In contrast, the positive control protease, which dispersed 80% of the SRB biofilm formed in modified Baar’s medium, dispersed only about 40% of the biofilm grown in M9G.

### Genetic basis of PA14 supernatant dispersal

To determine if there was a genetic component to the dispersal of the SRB cells upon the addition of the *P*. *aeruginosa* supernatants, gene expression levels of dispersed *D*. *vulgaris* biofilm cells after contact with wild-type PA14 supernatants were compared to non-dispersed *D*. *vulgaris* cells contacted *rhlA* PA14 supernatants, via RNA-seq. Hence, the role of rhamnolipids in SRB biofilm dispersal was analyzed.

The genes that had significant differences in expression levels in dispersed cells are shown in Supplementary Table 2 (induced genes) and Supplementary Table 3 (repressed genes). The gene expression levels were quantified as transcript per kilobases million (TPM) for each gene. Based on the results in Supplementary Table 2 and Supplementary Table 3, we cloned one gene (*dvua0018)* that was induced and four genes (*dvua0034*, *dvua0066*, *dvua0084*, and *dvu3313*) that were repressed and tested whether production of the proteins impact SRB biofilm formation. These genes were chosen based on these criteria: (i) their high differential expression, (ii) their significant TPM values (which indicates they are actively expressed), and (iii) their position in the genome which indicates they are not in the same operon so we avoided polar affects. To investigate the importance of these SRB proteins in biofilm formation, we utilized a promoter that is induced solely in *D*. *vulgaris* biofilms (P_*dvu0304*_, unpublished). We found DVUA0018, DVUA0034, DVUA0066, and DVUA0084 significantly affect biofilm formation, with production of DVUA0066 (a likely phospholipase) reducing biofilm formation 5.6-fold (Fig. 4d). These four proteins are found on the megaplasmid; hence, we identified specific proteins on this megaplasmid that influence biofilm formation.

## Discussion

We have demonstrated that the supernatants of PA14 disperse *D*. *vulgaris* biofilms effectively and that the biochemical basis of this dispersal is the presence rhamnolipids. The PA14 supernatants dispersed the biofilm better than protease, and they are versatile because they also dispersed *D*. *desulfuricans*, *E*. *coli* MG1655, and *S*. *aureus* biofilms. This is significant since the matrix of *D*. *desulfuricans*, *E*. *coli*, and *S*. *aureus* are primarily proteins,^12,13^ carbohydrates,^44^ and some extracellular genomic DNA,^14^ respectively, which indicates that rhamnolipids are a powerful and general approach for removing biofilms. Though the supernatant from the *rhlR* mutant did not disperse biofilms of *S*. *aureus* as well as the PA14 wild-type supernatants, it dispersed about 70% of the biofilm. Hence, there may be other compounds in the PA14 supernatants beyond rhamnolipids that disperse *S*. *aureus* biofilms.

The rhamnolipid standard did not disperse the biofilm as well as the rhamnolipids in the PA14 WT supernatant, possibly because the ratios of the rhamnolipids are critical for biofilm dispersal activity. For example, the ratio between di-rhamnolipids and mono-rhamnolipids affects the mixture properties (e.g., foam thickness, surface electric parameters)^45^ and changes the emulsification index and antimicrobial properties.^46^ In addition, the rhamnolipid standard was not pure (90%), and the growth condition used to produce the rhamnolipids is unknown. We also surmise that purifying the rhamnolipids alters their biofilm dispersal properties.

Previously, it was reported that rhamnolipids from *P*. *aeruginosa* together with the QS signal 3oxoC12HSL work cooperatively to disperse *E*. *coli* biofilms^47^; however, we found that there was no need for 3oxoC12HSL for supernatants to disperse SRB since the supernatants from the *lasI* mutant were just as effective as those from the wild-type strain (Fig. 1b). Furthermore, adding either C4-HSL or 3oxoC12HSL had no effect on SRB biofilm dispersal by the wild-type PA14 supernatants (Fig. 1c). Therefore, dispersal of the *D*. *vulgaris* biofilm by rhamnolipids is not due to these QS signals from *P*. *aeruginosa*.

Based on the whole-transcriptome results (RNA-seq) comparing the expression levels of SRB genes from cells dispersed from biofilms (contacted with PA14 supernatants) vs. their expression in non-dispersed biofilm cells (contacted with PA14 *rhlA* supernatants that lack rhamnolipids), four proteins (DVUA0018, DVUA0034, DVUA0066, and DVUA0084) were found to impact SRB biofilm formation. Critically, all of them are encoded by the megaplasmid of *D vulgaris* and production of DVUA0066 reduced biofilm formation by over 5-fold. Hence, by studying the genetic basis of dispersal with rhamnolipids, we identified specific proteins of the megaplasmid involved in biofilm formation. Previously, the megaplasmid (but not specific proteins) has been shown to increase biofilm formation 300%.^5^ In summary, relatively inexpensive supernatants containing rhamnolipids may be used to disperse industrially-relevant biofilms as well as to provide insights into the genetic basis of biofilm formation.

## Methods

### Bacterial species and culture conditions

*Desulfovibrio vulgaris* and *Desulfovibrio desulfuricans* were grown anaerobically in modified Baar’s medium at 30 °C or minimal medium with 0.4% glucose (M9G)^48^. PA14 wild-type and its mutants were grown in lysogeny broth (LB)^49^ or M9G at 37 °C. Gentamicin (Gm) 15 μg/mL was used for culturing the PA14 mutants. *Escherichia coli* TG1, *Escherichia coli* MG1655, *Bacillus subtilis*, and *Pseudomonas fluorescens* were grown in LB at 37 °C. For the recombinant strains to maintain broad host vector plasmid pVLT33, 50 ng/μL kanamycin was added to LB for *E*. *coli* and 400 ng/μL G418 was added to modified Baar’s medium (ATCC medium no. 1249) with 0.025% sodium sulfide (as an oxygen scavenger) for *D*. *vulgaris*. All the species are shown in Supplementary Table 4. The PA14 *rhlA*, *rhlB*, *rhlI*, *rhlR*, and *lasI* mutants^50,51^ were verified via PCR using the primers in Supplementary Table 5.

### Cloning biofilm dispersal-related genes identified by RNA-seq

To investigate whether proteins encoded by the differentially-expressed genes in the RNA-seq data for SRB treated with *P*. *aeruginosa* supernatant samples could affect biofilm formation, *dvua0018*, *dvu3313*, *dvua0084*, *dvua0034* and *dvua0066* were cloned into broad-host range plasmid pVLT33^52^ under control of the biofilm-specific promoter P_*dvu0304*_ with ribosome binding site AAGGAG. The primers for construction pVLT33 derivatives are listed in Supplementary Table 5. *E*. *coli* TG1 was used for the constructions of all the plasmids. Correct construction of pVLT33 derivatives were confirmed by sequencing with primers pVLT33-SF and pVLT33-SB, which locate at the upstream and downstream of multiple cloning site on plasmid pVLT33 respectively.

To electroporate the pVLT33 derivatives into wild-type *D*. *vulgaris*, anaerobically, competent cells (turbidity ~0.3 at 600 nm) were prepared by washing twice with pre-chilled, sterile 10% glycerol. In the anaerobic chamber, plasmid DNA (0.5–1 µg) was added to competent cells (50 µL) by mixing gently, and the solution was transferred to a pre-chilled (0 °C), 1 mm electroporation cuvette (Fisherbrand cat #FB101, Fisher Scientific, USA). Electroporation was performed aerobically (conditions: 25 µF, 200 Ω, and 1.5 kV/cm). The cuvette was returned to the anaerobic chamber, and 1 mL of modified Baar’s medium was added immediately. The cells were mixed gently and transferred to a 1.5 mL Eppendorf tube where they were recovered overnight at 30 °C anaerobically. After the recovery, 50 µL was inoculated into 10 mL modified Baar’s medium (0.2% yeast extract) with G418 (400 µg/mL) or plated on modified Baar’s medium (0.2% yeast extract) 1% agar plate with G418 (400 µg/mL). Genomic DNA from 1 to 2 mL of culture or the colony was isolated using an UltraClean^@^ Microbial DNA isolation kit (MO BIO cat#12224, Qiagen, USA) for PCR verification. During the PCR verification of the transformation of the cloned genes *dvua0018*, *dvu3313*, *dvua0084*, *dvua0034*, and *dvua0066* into *D*. *vulgaris*, primers pVLT33-SB and dvua0018-*Bam*HI-R2, pVLT33-SB and dvu3313-*Bam*HI-R2, pVLT33-SB and dvua0084-*Bam*HI-R2, pVLT33-SB and dvua0034-R, and pVLT33-SB and dvua0066-R were used, respectively (Supplementary Table 5).

### PA14 supernatant preparation

*P*. *aeruginosa* PA14 wild-type and its isogenic mutants were grown in LB or LB with Gm15 μg/mL for the mutants. The overnight cultures (200 µL) were centrifuged, the cell pellets were resuspended in 50 mL of M9G, and cultured for 4–5 days. The supernatants were collected and filtered using a 0.22 µm filtration system. The samples were concentrated 4× using a speed vacuum.

### Biofilm dispersal assay using crystal violet

Biofilm formation was assayed in 96-well polystyrene plates using 0.1% crystal violet staining as described previously^53^ with some modifications. Diluted overnight cultures (150 µL) at an initial turbidity at 600 nm of 0.05 were inoculated into 96-well-plates with medium, and the bacteria were cultured for 24–48 h at 30 °C without shaking. The planktonic cells were removed from each well, and the biofilm was washed gently using phosphate buffered saline (PBS). The supernatant (150 µL, at the indicated concentrations), chemicals (including the positive control protease Savinase, 0.024 U), or PBS (as a control) were added to the wells, and the plate was incubated at 30 °C for 1–2 h. Then, the supernatants were removed from each well, and the remaining biofilm was washed gently using water. After the crystal violet was added to each well, the wells were rinsed and dried, and ethanol was added to dissolve the crystal violet. The total biofilm remaining in the samples were measured at 540 nm. *N*-butanoyl-L-homoserine lactone (C4-HSL, Cayman Chemical, Ann Arbor, MI, catalog number 10007898) or *N*-(3-oxododecanoyl)-homoserine lactone (3oxoC12HSL, Cayman Chemical, catalog number 10007895) were used at 50 µM in dimethylformamide (DMF); DMF was used as the solvent control. At least two independent cultures were used for each strain with three replicates for each culture.

### Cell viability

Cell viability was determined by staining SYTO9 and propidium iodide of the LIVE/DEAD BacLight Bacterial Viability Kit (Thermo Fisher Scientific, Waltham, MA). For planktonic cell viability, a *D*. *vulgaris* culture (1 mL) was centrifuged, 0.9 mL removed, and the remaining 0.1 mL (with the resuspended cell pellet) was contacted with 0.1 mL of *P*. *aeruginosa* PA14 supernatants (10×) for a final 5× supernatant concentration or contacted with 10 mM commercial rhamnolipids. The samples were incubated at 30 °C for 2 h anaerobically. PBS treatment (2 h) was used as a negative control, and ethanol (70%) treatment for 30 min was used as the positive control for cell death. Prior to staining, cells were washed once with 0.85% NaCl and resuspended in 0.1 mL of 0.85% NaCl. For *D*. *vulgaris* biofilm cell viability, biofilm cells were formed for 48 h in 96 well plates in 150 μL, washed with 150 μL of PBS buffer (pH 7.4), and contacted with 150 μL of *P*. *aeruginosa* PA14 supernatants (4×) and 10 mM commercial rhamnolipids for 2 h anaerobically. Then the liquids were removed and 150 μL of PBS buffer was added. The cells were stained at room temperature for 15 min. The samples were observed under a fluorescence microscope (Zeiss Axioscope.A1). At least three different areas were observed, and two independent cultures were tested.

### Confocal microscopy

Diluted overnight cultures (300 µL) at an initial turbidity at 600 nm of 0.1 were inoculated into an 8 chamber cell culture slide (Dot Scientific, Inc, Burton, MI) with modified Baar’s medium, and the bacteria were cultured for 48 h at 30 °C without shaking. The planktonic cells were removed from each well, and the biofilm was washed gently using PBS. Supernatants or PBS was added to the chambers, and the slide was incubated at 30 °C for 20 min. The supernatants or PBS were removed from the chamber. Biofilm cells were washed with 300 µL PBS, then stained with SYTO 9 (5 µL/mL) in the dark for 1 h. Confocal microscopy images were taken using a PlanApo60×/1.4 oil objective lens with Olympus Fluoview 1000 confocal laser microscope. The biofilm structure images were performed using IMARIS software (Bitplane, Zurich, Switzerland), and average biomass and thickness were obtained using COMSTAT image-processing software^54^. At least three different areas were observed, and two independent cultures were tested.

### Mass spectrometric analysis of supernatants

Mass spectrometric analysis was performed on the concentrated supernatants by using a Waters Q-TOF Premier quadrupole/time-of-flight (TOF) mass spectrometer (Waters Corporation (Micromass Ltd.), Manchester, UK). Operation of the mass spectrometer was performed using MassLynx™ software Version 4.1 (http://www.waters.com). Samples were introduced into the mass spectrometer using a Waters 2695 high performance liquid chromatograph. The samples were analyzed using flow injection analysis (FIA), in which the sample is injected into the mobile phase flow and passes directly into the mass spectrometer, where the analytes are ionized and detected. The mobile phase used was 90% acetonitrile (LC-MS grade), and 10% aqueous 10 mM ammonium acetate. The flow rate was 0.15 mL/min. The nitrogen drying gas temperature was set to 300 °C at a flow of 7 L/min. The capillary voltage was 2.8 kV. The mass spectrometer was set to scan from 100 to 1000 m/z in negative ion mode, using electrospray ionization (ESI). The rhamnolipids standard from *P*. *aeruginosa* (90%) was purchased from Sigma-Aldrich (St. Louis, MO). Rhamnolipid peaks were identified as disclosed previously^55^.

### Sample preparation for RNA-seq

*D*. *vulgaris* was grown anaerobically at 30 °C for 24 h in 400 mL of modified Baar’s medium with 10 g of glass wool to promote biofilm formation. The glass wool was rinsed once in PBS to remove planktonic cells. The glass wool with biofilm cells was incubated in 200 mL of PA14 wild-type or *rhlA* mutant supernatants (1×) for 30 min anaerobically. The glass wool from the sample with the PA14 wild-type supernatant was removed, and the dispersed biofilm cells in the supernatant were harvested by centrifugation at 4 °C. Since the biofilm cells in the glass wool from the sample with the *rhlA* mutant supernatant was not dispersed, cells were harvested from the glass wool for RNA-seq. The glass wool was gently washed twice in 200 ml of cold 0.85% NaCl buffer and sonicated for 2 min in 200 mL of cold 0.85% NaCl with 2 mL of RNA later (Thermo Fisher Scientific, Waltham, MA) by using an ultrasonic water bath. The non-dispersed biofilm cells were centrifuged at 8000*g* at 4 °C. The biofilm cell pellets were washed in 4 mL of cold RNA later and kept at −80°C for RNA isolation. RNA was extracted using the High Pure RNA isolation kit (Roche, Basel, Switzerland). The experiments were performed with two independent cultures. The RNA samples were sent to the Genome Sciences Facility at Pennsylvania State College of Medicine, Hershey, PA to evaluate RNA quality and to perform RNA-seq.

## Electronic supplementary material

Supplemental Information

## Data Availability

The authors declare that data supporting the findings of this study are available within the paper, and the raw RNA sequencing reads have been submitted to the NCBI Sequence Read Archive (SRA) database (http://www.ncbi.nlm.nih.gov) under SRA accession no. SRP154476.

## Electronic supplementary material

**Supplementary information** accompanies the paper on the *npj Biofilms and Microbiomes* website (10.1038/s41522-018-0066-1).
