# Supplementary material for: Rhamnolipids from Pseudomonas aeruginosa disperse the biofilms of sulfate-reducing bacteria
Source: NPJ Biofilms Microbiomes. 2018 Oct 3;4:22. doi: 10.1038/s41522-018-0066-1 (PMC6170446; doi:10.1038/s41522-018-0066-1)
Supplement: Supplementary file 1 — Supplemental Information [file 41522_2018_66_MOESM1_ESM.pdf]

SUPPLEMENTARY INFORMATION

Rhamnolipids from *Pseudomonas aeruginosa* Disperse the Biofilms of Sulfate-Reducing Bacteria

Thammajun L. Wood[1], Ting Gong[1], Lei Zhu[1], James Miller[3], Daniel S. Miller[4], Bei Yin[4], and Thomas K. Wood[1,2,3*]

[1]Department of Chemical Engineering, [2]Department of Biochemistry and Molecular Biology, and the [3]Huck Institutes of the Life Sciences, Pennsylvania State University, University Park, Pennsylvania, 16802

[4]Dow Chemical Company, Collegeville, PA, 19426

*Correspondence should be addressed to T. K. W. (tuw14@psu.edu)

Keywords: rhamnolipids, biofilm dispersal, *Pseudomonas aeruginosa,* sulfate-reducing bacteria

Running head: Rhamnolipids disperse SRB biofilms

Supplementary Table 1. Rhamnolipids in *P. aeruginosa* PA14 4X concentrated supernatants and in the 10 mM commercial rhamnolipid standard. Peak areas are indicated from the mass spectrometry chromatograms.

Rhamnolipids[53]	Ion (m/z)	PA 14 WT	Standard
Rha-C_{10}-C_{10}	503.3	31	6374
Rha-C_{10}-C_{10}/Rha-C_{12}-C_{10}	531.3	3	294
Rha-Rha-C_{10}-C_{10}	649.3	37	3465
Rha-C_{10}-C_{12}/Rha-C_{12}-C_{10}	677.3	8	178

Supplementary Table 2. Induced genes in dispersed biofilm cells of *D. vulgaris* compared with non-dispersed biofilm cells. Bold genes were cloned and tested for their impact on SRB biofilm formation.

**Gene ID**	**Average TPM* non dispersal cells**	**Average TPM* dispersal cells**	**Fold change**	**Protein**
**Hypothetical proteins**				
dvua0018	2888	25892	8.97	hypothetical protein DVUA0018 (plasmid)
dvua0030	761	3715	4.88	hypothetical protein DVUA0030 (plasmid)
dvua0031	107	6599	61.66	hypothetical protein DVUA0031 (plasmid)
dvua0108	35220	108654	3.09	hypothetical protein DVUA0108 (plasmid)
dvua0126	0	5439	∞	hypothetical protein DVUA0126 (plasmid)
dvua0131	974	3808	3.91	hypothetical protein DVUA0131 (plasmid)
dvu0251	320	1021	3	hypothetical protein DVU0251
dvu0603	18	126	7	hypothetical protein DVU0603
dvu0994	190	710	4	hypothetical protein DVU0994
dvu1670	34	227	7	hypothetical protein DVU1670
dvu1966	5	354	77	hypothetical protein DVU1966
dvu2105	1501	6847	5	hypothetical protein DVU2105
dvu2108	1284	6262	5	hypothetical protein DVU2108
dvu2803	921	2837	3	hypothetical protein DVU2803
**Transcriptional regulators**				
dvu0629	28	180	6	TetR family transcriptional regulator
dvu1144	22	154	7	Cro/CI family transcriptional regulator
dvu1572	54	309	6	CarD family transcriptional regulator
**ATP synthase**				
dvu0776	0	80	∞	F0F1 ATP synthase subunit gamma
dvu0777	65	340	5	F0F1 ATP synthase subunit alpha
**Other highly expressed and induced genes**				
dvua0012	238	1368	5.76	nitrogenase molybdenum-iron protein subunit alpha (plasmid)
dvua0019	3024	8252	2.73	type II DNA modification methyltransferase (plasmid)
dvua0091	37752	105923	2.81	catalase (plasmid)
dvu0557	78	515	7	T_locus_tag_DVU_0557
dvu1109	76	356	5	ATPase
dvu1157	43	489	11	sensory box histidine kinase
dvu1290	0	41	∞	nitrate reductase subunit gamma
dvu1316	277	1078	4	30S ribosomal protein S14
dvu1404	43	273	6	radical SAM domain-containing protein
dvu1580	88	398	5	ribose 5-phosphate isomerase
dvu1876	0	162	∞	DnaJ protein
dvu2421	68	421	6	4-oxalocrotonate tautomerase
dvu2971	123	391	3	glycosyl transferase family protein

*TPM is transcripts per kilobase million and was used to directly compare the proportion of reads that map to a gene in each sample.

Supplementary Table 3. Repressed genes in dispersed biofilm cells of *D. vulgaris* compared with non-dispersed biofilm cells. Bold genes were cloned and tested for their impact on SRB biofilm formation.

Gene	Average TPM non dispersal cells	Average TPM dispersal cells	Fold change	Protein
**Hypothetical proteins**				
dvua0001	2865	0	-∞	hypothetical protein DVUA0001 (plasmid)
dvua0017	3384	0	-∞	hypothetical protein DVUA0017 (plasmid)
dvua0035	3684	0	-∞	hypothetical protein DVUA0035 (plasmid)
dvua0055	5800	0	-∞	hypothetical protein DVUA0055 (plasmid)
dvua0060	4481	0	-∞	hypothetical protein DVUA0060 (plasmid)
dvua0107	4993	0	-∞	hypothetical protein DVUA0107 (plasmid)
dvua0128	4832	0	-∞	hypothetical protein DVUA0128 (plasmid)
dvua0147	6631	279	-23.79	hypothetical protein DVUA0147 (plasmid)
dvua0152	1970	0	-∞	hypothetical protein DVUA0152 (plasmid)
dvu3268	181	0	-∞	hypothetical protein DVU3268
**Transcriptional regulators**				
dvua0084	4851	0	-∞	AbrB family transcriptional regulator
dvu3313	109	0	-∞	LysR family transcriptional regulator
**Other highly expressed and repressed genes**				
dvua0008	8149	220	-37.03	nitrogenase molybdenum-iron cofactor biosynthesis protein NifN (plasmid)
dvua0010	591	0	-∞	
dvua0013	4019	0	-∞	nitrogen regulatory protein P-II (plasmid)
dvua0014	397	0	-∞	
dvua0015	1827	0	-∞	
dvua0034	5626	1428	-3.94	
dvua0038	3037	0	-∞	capsular polysaccharide transport protein
dvua0046	8737	0	-∞	glycosyl transferase, group 2 family protein
dvua0051	9402	107	-87.71	
dvua0053	4024	0	-∞	
dvua0066	3288	1266	-2.60	
dvua0078	872	0	-∞	
dvua0083	1333	0	-∞	
dvua0130	1694	0	-∞	CRISPR-associated Csd1 family protein
dvua0134	873	9	-102.44	
dvua0135	601	0	-∞	
dvua0141	1052	0	-∞	
dvua0144	4435	0	-∞	
dvua0151	6013	0	-∞	DNA-binding protein (plasmid)
dvua0016	9919	734	-13.51	homocitrate synthase (plasmid)
dvu0115	261	4	-74	shikimate 5-dehydrogenase
dvu0862	345	8	-43	bifunctional flagellar protein FliS/hypothetical protein
dvu2749	285	6	-52	precorrin-6Y C5,15-methyltransferase (decarboxylating)
dvu2906	277	7	-40	umuC protein
dvu3303	177	0	-∞	ATP-dependent protease La
dvu3337	182	0	-∞	K+-transporting ATPase subunit C

*TPM is transcripts per kilobase million and was used to directly compare the proportion of reads that map to a gene in each sample.

Supplementary Table 4. Bacterial species and plasmids used in this study. Gm^R and $G418^R$ denote gentamicin and geneticin resistance, respectively.

Species	Description	Source
**Species**		
E. coli TG1	*supE thi-1 Δ(lac-proAB) Δ(mcrB-hsdSM)5,* $(r_K^- m_K^-)$ F' [*traD36 proAB*$^+$ *lacI*^{q} *lacZΔM15*]	Jeremy Minshull
E. coli MG1655	wild-type	Frederick Blattner
D. vulgaris	wild-type	ATCC 29579
D. desulfuricans	wild-type	DSM 12129
P. aeruginosa PAO1	wild-type	Tim McDermott
P. aeruginosa PA14	ATCC 29579, wild-type	Frederick Ausubel
P. aeruginosa PA14 *Δ lasI*	PA14_45940, Mar2xT7 transposon insertion, Gm^R	[54]
P. aeruginosa PA14 *Δ lasB*	PA14_16250, Mar2xT7 transposon insertion, Gm^R	[54]
P. aeruginosa PA14 *Δ pelA*	PA14_24480, Mar2xT7 transposon insertion, Gm^R	[54]
P. aeruginosa PA14 *Δ phzM*	PA14_09490, Mar2xT7 transposon insertion, Gm^R	[54]
P. aeruginosa PA14 *Δ phzS*	PA14_09400, Mar2xT7 transposon insertion, Gm^R	[54]
P. aeruginosa PA14 *Δ pvdF*	PA14_33700, Mar2xT7 transposon insertion, Gm^R	[54]
P. aeruginosa PA14 *Δ pchE*	PA14_09270, Mar2xT7 transposon insertion, Gm^R	[54]
P. aeruginosa PA14 *Δ lecA*	PA14_31290, Mar2xT7 transposon insertion, Gm^R	[54]
P. aeruginosa PA14 *Δ toxA*	PA14_49560, Mar2xT7 transposon insertion, Gm^R	[54]
P. aeruginosa PA14 *Δ sodM*	PA14_58000, Mar2xT7 transposon insertion, Gm^R	[54]
P. aeruginosa PA14 *Δ rhlA*	*rhlA*::Gm, Gm^R	[55]
P. aeruginosa PA14 *Δ rhlB*	PA14_19110, Mar2xT7 transposon insertion, Gm^R	[54]
P. aeruginosa PA14 *Δ rhlI*	PA14_19130, Mar2xT7 transposon insertion, Gm^R	[54]
P. aeruginosa PA14 *Δ rhlR*	PA14_19120, Mar2xT7 transposon insertion, Gm^R	[54]
P. fluorescens	wild-type	Richard Frazee
S. aureus	ATCC 29213, wild-type	Kenneth Urish
**Plasmids**		
pVLT33-$P_{dvu0304}$	broad host plasmid pVLT33 with promotor $P_{dvu0304}$ from *D. vulgaris* (ATCC 29579), $G418^R$	This study
pVLT33-$P_{dvu0304}$-*dvu3313*	pVLT33 containing gene *dvu3313* with promotor $P_{dvu0304}$, $G418^R$	This study
pVLT33-$P_{dvu0304}$-*dvua0018*	pVLT33 containing gene *dvua0018* with promotor $P_{dvu0304}$, $G418^R$	This study
pVLT33-$P_{dvu0304}$-*dvua0034*	pVLT33 containing gene *dvua0034* with promotor $P_{dvu0304}$, $G418^R$	This study
pVLT33-$P_{dvu0304}$-*dvua0066*	pVLT33 containing gene *dvua0066* with promotor $P_{dvu0304}$, $G418^R$	This study
pVLT33-$P_{dvu0304}$-*dvua0084*	pVLT33 containing gene *dvua0084* with promotor $P_{dvu0304}$, $G418^R$	This study

Supplementary Table 5. Primers used in this study. Forward primers are labeled with F, and rear primers are labeled with R.

**Name**	**Sequence**
rhlA F2	ATGGCCGCTGAGTTACTTGTCT
rhlA R2	CAGGCCGATGAAGGGAAATACGT
rhlB F	AAGACCCGGCCTGGCCGGGC
rhlB R	ATTGCAGTAAGCCCTGATCG
rhlI F	AGAGGGCCCAGGAGTATCA
rhlI R	GGCTGACGACCTCACACC
rhlR F	GTGATGCATTTTATCGATC
rhlR R	AACCTGCCAGATCTGGTAGGGC
lasI F	TGGAGGAAGTGAAGATGATCGT
lasI R	ACAGGTCCCCGTCATGAAAC
*Hin*dIII site-PF1	CCCAAGCTTTCCGTGTTGACATTGATTTTCG
dvua0084R1	GAAGTCTGCTTGAGCACGACGGGCTCCTTGGTTTG
dvua0084F2	CAAACCAAGGAGCCCGTCGTGCTCAAGCAGACTTCCAC
dvua0084-*Bam*HI-R2	TTTCGCGGATCCTCAGTCCGTTTCGCCCTGTGGCAA
dvu3313R1	AGTTGACGGAGTTCCATGACGGGCTCCTTGGTTTG
dvu3313F2	CAAACCAAGGAGCCCGTCATGGAACTCCGTCAACTCCG
dvu3313-*Bam*HI-R2	TTTCGCGGATCCTCACCCTTCCATCCCGAAGGGCCG
dvua0018R1	TCTTGGGTTTGATTCAAGACGGGCTCCTTGGTTTG
dvua0018F2	CAAACCAAGGAGCCCGTCTTGAATCAAACCCAAGATAC
dvua0018-*Bam*HI-R2	TTTCGCGGATCCTCAGAAGAAACTCGAGAGATC
dvua0034-F	CGCGGATCCATGCTCGCCATCGCCGACAAGCGCC
dvua0034-R	CCGGAATTCTCACTCGGCAGGCAGCAGGAAGTCC
dvua0066-F	CGCGGATCCATGTACCGCAATATCGTGTTCAAGG
dvua0066-R	CCGGAATTCTCATGCCATGCCCCCATCACGGTAG
pVLT33-SB	ACTAAGCTGATCCGGTGGATG
pVLT33-SF	GAAATGAGCTGTTGACAATTAATCA

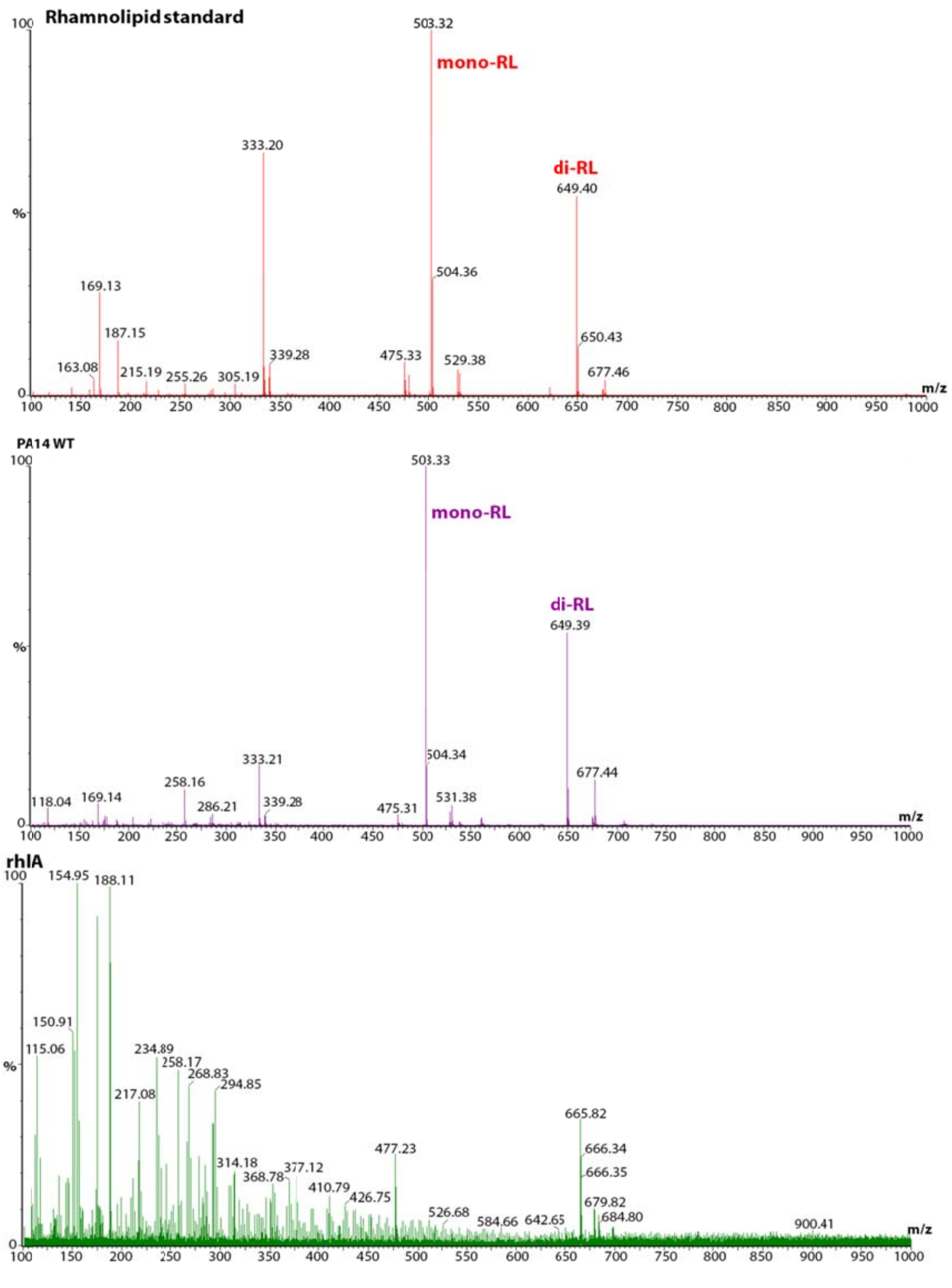

Supplementary Figure 1. Detection of rhamnolipids in the *P. aeruginosa* PA14 supernatants via mass spectrometry. *P. aeruginosa* wild-type and the *rhlA* mutant were grown planktonically for 4 days in M9G medium at 37 ºC. The supernatants were collected, filtered, and concentrated to 4X. The Y axis indicates the percentage each peak is relative to the maximum peak size. Mono-rhamnolipids (mono-RL) and di-rhamnolipids (di-RL) were detected in the wild-type supernatant (middle) and the commercial standards (top) but not in the *rhlA* supernatant (bottom).
